# Clinical Predictors of Non-Diabetic Kidney Disease in Patients with Diabetes: Insights from a Biopsy-Proven Cohort

**DOI:** 10.3390/jcm15114346

**Published:** 2026-06-04

**Authors:** To-Pang Chen, Shang-Feng Tsai

**Affiliations:** 1 Division of Endocrinology and Metabolism, Show Chwan Memorial Hospital, 542 Chung-Shan Road (Section 1), Changhua 50008, Taiwan; 2 Division of Nephrology, Department of Internal Medicine, Taichung Veterans General Hospital, Taichung 407219, Taiwan; 3 Department of Post-Baccalaureate Medicine, College of Medicine, National Chung Hsing University, Taichung 402202, Taiwan; 4 Division of Clinical Informatics, Department of Digital Medicine, Taichung Veterans General Hospital, Taichung 407219, Taiwan

**Keywords:** diabetes mellitus, non-diabetic kidney disease, renal biopsy, clinical predictor, chronic kidney disease

## Abstract

**Background:** Distinguishing diabetic nephropathy (DN) from non-diabetic kidney disease (NDKD) in patients with diabetes remains clinically challenging, particularly when renal biopsy is not routinely performed. We aimed to identify clinical predictors of biopsy-proven NDKD. **Methods:** We conducted a retrospective cohort study of patients with type 2 diabetes who underwent native kidney biopsy at a tertiary referral center. Patients were classified as DN alone, mixed DN with NDKD, or pure NDKD. Baseline clinical and laboratory variables were analyzed. Logistic regression models were used to identify factors associated with NDKD. **Results:** Among 664 patients, 18.7% had DN alone, 27.0% had mixed lesions, and 54.3% had pure NDKD. In multivariable analysis, higher HbA1c, lower body mass index, lower low-density lipoprotein cholesterol, and higher urine albumin-to-creatinine ratio were independently associated with NDKD. For pure NDKD, lower HbA1c, lower serum albumin, lower body mass index, higher IgA levels, and absence of hypertension were significant predictors. **Conclusions:** NDKD is common among patients with diabetes undergoing biopsy and can be partially predicted using routinely available clinical parameters. These findings may aid in identifying patients who could benefit from timely renal biopsy and individualized management.

## 1. Introduction

Chronic kidney disease (CKD) represents a major global health burden [1]. Taiwan has one of the highest incidences and prevalences of end-stage kidney disease (ESKD) worldwide, with dialysis prevalence exceeding 3500 patients per million population according to national registry data [2,3,4]. Despite advances in supportive care, CKD remains largely irreversible, and progression to ESKD continues to impose substantial clinical and economic challenges. Among all etiologies, type 2 diabetes mellitus (DM) is the leading cause of CKD and ESKD [5], accounting for a large proportion of cases globally.

Diabetic nephropathy (DN) has traditionally been considered the predominant cause of kidney dysfunction in patients with diabetes and is often diagnosed based on clinical features without histological confirmation [6]. However, not all kidney disease in patients with diabetes is attributable to DN [7]. A substantial proportion of patients may have non-diabetic kidney disease (NDKD) [7], either as an isolated condition (36%) [8] or superimposed on DN (27%) [8]. Distinguishing DN from NDKD is clinically important, as the underlying etiology may influence prognosis and therapeutic strategies.

Several clinical features [9], such as the absence of diabetic retinopathy, presence of hematuria, or discordance between proteinuria and renal function, have been proposed to suggest NDKD. However, these indicators lack sufficient sensitivity and specificity, and reliance on clinical judgment alone may result in misclassification. Renal biopsy remains the gold standard for differentiating DN from NDKD, but its invasive nature limits routine use in clinical practice. Given these challenges, there is a critical need to identify reliable clinical predictors that can help stratify the likelihood of NDKD and guide decision-making regarding renal biopsy. Leveraging a large biopsy-based cohort from a tertiary referral center in Taiwan, we aimed to identify clinical factors associated with biopsy-proven NDKD in patients with type 2 diabetes.

## 2. Materials and Methods

### 2.1. Data Source and Study Population

We conducted a retrospective cohort study using the renal biopsy database of Taichung Veterans General Hospital, a high-volume tertiary referral center in Taiwan. The database comprises more than 9000 native kidney biopsy cases collected over the study period. For the present analysis, we included adult patients with a diagnosis of DM who underwent native kidney biopsy. Patients who underwent kidney transplant (allograft) biopsy were excluded. Clinical data, including baseline characteristics and laboratory parameters at the time of biopsy, were extracted from the institutional electronic health record system through a standardized extract–transform–load process. The primary outcome was biopsy-proven NDKD, either as isolated NDKD or as mixed lesions coexisting with DN. The objective of this study was to identify baseline clinical variables associated with NDKD. This study was approved by the Human Research Review Committee of Taichung Veterans General Hospital (approval number CE24589B). All procedures were conducted in accordance with institutional review board regulations and relevant ethical guidelines.

Kidney biopsy was generally considered in patients with diabetes presenting with atypical clinical features inconsistent with classic diabetic nephropathy, including abrupt onset nephrotic-range proteinuria, rapid deterioration of kidney function, active urinary sediment, hematuria, unexplained decline in eGFR, or clinical suspicion of superimposed glomerular disease. Biopsy decisions were made by treating nephrologists in accordance with routine clinical practice and contemporary KDIGO recommendations [6]. All kidney biopsy specimens were reviewed and interpreted by experienced renal pathologists at our institution using standard nephropathological evaluation, including light microscopy, immunofluorescence, and electron microscopy when clinically indicated.

### 2.2. Baseline Data Collection

Baseline demographic, clinical, laboratory, immunologic, urinary, and comorbidity data at the time of renal biopsy were collected for association analyses. Demographic variables included age (years), male sex (%), and body mass index (BMI, kg/m^2^). Hematologic parameters included hemoglobin (g/dL), white blood cell count (WBC, ×10^9^/L), and platelet count (×10^9^/L). Renal function parameters included serum creatinine (mg/dL), estimated glomerular filtration rate (eGFR, mL/min/1.73 m^2^), and blood urea nitrogen (BUN, mg/dL). Biochemical variables included sodium (mmol/L), potassium (mmol/L), and serum albumin (g/dL). Metabolic parameters included fasting glucose (mg/dL), glycated hemoglobin (HbA1c, %), low-density lipoprotein cholesterol (LDL, mg/dL), and uric acid (mg/dL). Immunologic markers included anti-neutrophil cytoplasmic antibody (ANCA, positive/negative), anti-double-stranded DNA (dsDNA, positive/negative), complement C3 (mg/dL), and complement C4 (mg/dL). Urinary parameters included urine albumin-to-creatinine ratio (UACR, mg/g), urine protein-to-creatinine ratio (UPCR, mg/g), and hematuria (presence of urine red blood cells, yes/no). Baseline comorbidities included hypertension, systemic lupus erythematosus, and asthma (all recorded as yes/no). All variables were obtained from the electronic health record system at the time of biopsy.

### 2.3. Definition of Non-Diabetic Kidney Disease and Outcomes

NDKD was defined as biopsy-proven or clinically established renal diseases not attributable to DN. These included ANCA-associated rapidly progressive glomerulonephritis, anti–glomerular basement membrane disease, immune complex–mediated glomerulonephritis, pigmented cast nephropathy, monoclonal gammopathy–related kidney disease, membranoproliferative glomerulonephritis, thrombotic microangiopathy, hypertensive nephrosclerosis, acute or chronic pyelonephritis, cryoglobulinemic glomerulonephritis, IgG4-related kidney disease, chronic glomerulonephritis, myoglobin cast nephropathy, renal amyloidosis, focal segmental glomerulosclerosis (primary or secondary), membranous nephropathy, lupus nephritis, post-infectious glomerulonephritis, IgA nephropathy, minimal change disease, and isolated interstitial nephritis. Non-specific pathological findings, including acute tubular injury, acute tubular necrosis, or other ill-defined diagnoses, were excluded.

Based on renal biopsy findings, patients were categorized into three groups: (1) DN alone, (2) mixed lesions consisting of DN coexisting with NDKD, and (3) pure NDKD without evidence of DN. For patients classified as having NDKD, detailed clinical information—including laboratory data, medication exposure, and underlying comorbidities—was reviewed to establish the specific diagnosis. The primary analysis evaluated factors associated with the presence of NDKD, defined as groups 2 and 3 combined versus group 1. A secondary analysis was performed to identify factors associated with pure NDKD (group 3) compared with the remaining groups.

### 2.4. Statistical Analysis

Continuous variables are presented as mean ± standard deviation or median (interquartile range), as appropriate, and categorical variables are expressed as counts and percentages. Comparisons among the three groups were performed using one-way analysis of variance (ANOVA) or the Kruskal–Wallis test for continuous variables and the chi-square test for categorical variables.

To identify factors associated with NDKD, univariate logistic regression analyses were first performed for each baseline variable. Variables with a *p*-value < 0.10 in univariate analysis or those considered clinically relevant were included in the multivariable logistic regression model. A stepwise selection approach was used to identify independent predictors while minimizing overfitting. Collinearity among covariates was assessed prior to model construction, and variables with significant multicollinearity were excluded.

The primary analysis evaluated predictors of NDKD (groups 2 and 3 combined versus group 1), and the secondary analysis focused on predictors of pure NDKD (group 3 versus groups 1 and 2). Adjusted odds ratios (aORs) with 95% confidence intervals (CIs) were reported. A two-sided *p*-value < 0.05 was considered statistically significant. Standardized mean differences (SMDs) were calculated to quantify the magnitude of between-group differences, with an SMD > 0.3 considered clinically meaningful.

## 3. Results

### 3.1. Study Population

**Figure 1:**
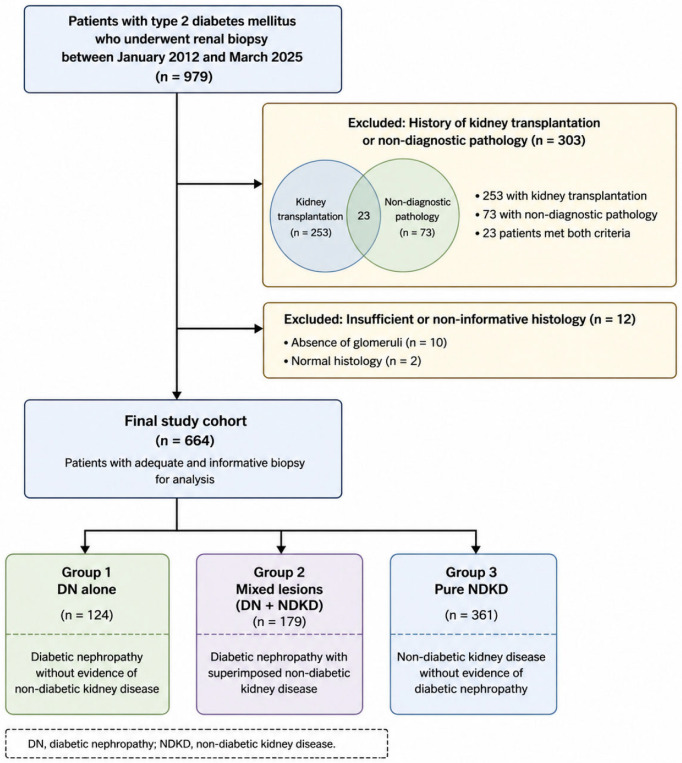
Study Population Selection Flowchart.

Between January 2012 and March 2025, a total of 979 patients with type 2 DM who underwent renal biopsy were screened for inclusion (Figure 1). Of these, 303 patients were excluded due to a history of kidney transplantation (n = 253) or non-diagnostic pathology (n = 73), with an overlap of 23 patients meeting both criteria. An additional 12 patients were excluded because of insufficient or non-informative histology, including absence of glomeruli (n = 10) or normal histology (n = 2). The final study cohort consisted of 664 patients. Based on renal biopsy findings, patients were classified into three groups: DN alone (group 1, n = 124), mixed lesions consisting of DN with superimposed NDKD (group 2, n = 179), and pure NDKD without evidence of DN (group 3, n = 361).

### 3.2. Baseline Characteristics

Baseline characteristics were generally comparable across the three groups (Table 1, and Supplementary Table S1 and Figure S1). Age and sex distribution did not differ significantly, whereas body mass index was modestly higher in the DN group. Hemoglobin levels were lower in the DN and mixed groups, while other hematologic parameters were similar. Renal function showed clear differences, with higher serum creatinine and lower eGFR in the DN group, whereas the NDKD group had better preserved renal function. Serum albumin was lowest in the mixed group. Metabolic parameters differed significantly, with lower fasting glucose and HbA1c observed in the NDKD group, while lipid levels, particularly low-density lipoprotein cholesterol, were higher in the mixed and NDKD groups. Immunologic abnormalities, including higher anti-double-stranded DNA positivity and lower complement C4 levels, were more frequently observed in the NDKD group. Urinary findings demonstrated more severe proteinuria in the mixed group, whereas hematuria was more prominent in the NDKD group. Among comorbidities, hypertension was most prevalent in the DN group, while systemic lupus erythematosus was more common in the NDKD group.

SMD analysis further highlighted clinically meaningful imbalances between DN and NDKD (Supplementary Figure S1). HbA1c showed the largest difference, followed by complement C4, renal function parameters (eGFR and creatinine), and immunologic markers, including anti-double-stranded DNA and lupus. Albumin and proteinuria (UPCR) also demonstrated moderate differences, whereas most other variables showed minimal imbalance. These findings indicate that metabolic, renal, and immunologic factors contribute to the differentiation between DN and NDKD and are consistent with the multivariable analysis.

### 3.3. Logistic Regression Analysis for Non-Diabetic Kidney Disease (Groups 2 and 3)

The most common non-diabetic kidney diseases identified in the cohort included primary focal segmental glomerulosclerosis (FSGS, 21.0%), IgA nephropathy (20.5%), membranous nephropathy (17.6%), lupus nephritis (11.4%), minimal change disease (9.4%), ANCA-associated glomerulonephritis (9.1%), crescentic glomerulonephritis (6.8%), post-infectious glomerulonephritis (3.0%), and monoclonal gammopathy–related kidney disease/multiple myeloma-associated nephropathy (1.1%).

Univariate and multivariable logistic regression analyses for NDKD (groups 2 and 3) are presented in Table 2. In univariate analysis, several clinical and laboratory variables were significantly associated with NDKD. Higher BMI (cOR 1.05, 95% CI 1.00–1.10, *p* = 0.037), lower hemoglobin (cOR 0.85, 95% CI 0.80–0.91, *p* < 0.001), higher serum creatinine (cOR 1.12, 95% CI 1.06–1.17, *p* < 0.001), and lower eGFR (cOR 0.99, 95% CI 0.98–0.99, *p* < 0.001) were associated with NDKD. Metabolic parameters, including fasting glucose (cOR 1.01, 95% CI 1.01–1.02, *p* < 0.001) and HbA1c (cOR 2.03, 95% CI 1.64–2.51, *p* < 0.001), also showed strong associations. Higher potassium levels (cOR 1.41, 95% CI 1.11–1.80, *p* = 0.005) and uric acid (cOR 0.87, 95% CI 0.80–0.95, *p* = 0.001) were also associated with NDKD. Immunologic markers, including ANCA (cOR 0.31, 95% CI 0.14–0.69, *p* = 0.003), dsDNA (cOR 0.35, 95% CI 0.22–0.54, *p* < 0.001), complement C3 (cOR 1.01, 95% CI 1.00–1.01, *p* = 0.012), and complement C4 (cOR 1.04, 95% CI 1.02–1.06, *p* < 0.001), were also significant.

In multivariable analysis, several variables remained independently associated with NDKD. Increasing age was inversely associated with NDKD (aOR 0.93, 95% CI 0.88–0.99, *p* = 0.021). BMI also demonstrated an inverse association after adjustment (aOR 0.81, 95% CI 0.70–0.95, *p* = 0.008). HbA1c remained a strong independent predictor (aOR 3.68, 95% CI 1.75–7.72, *p* = 0.001). Low-density lipoprotein cholesterol was inversely associated with NDKD (aOR 0.98, 95% CI 0.97–0.99, *p* = 0.002). UACR remained independently associated with the outcome (aOR 1.00038, 95% CI 1.0001–1.001, *p* = 0.007), whereas UPCR did not reach statistical significance after adjustment (aOR 0.99984, 95% CI 0.9997–1.0000, *p* = 0.061).

Overall, metabolic factors, particularly HbA1c, along with renal injury markers such as UACR and selected demographic variables, were independently associated with NDKD. In contrast, immunologic markers and comorbid conditions, although significant in univariate analysis, were not independently associated after multivariable adjustment.

### 3.4. Logistic Regression Analysis for Pure Non-Diabetic Kidney Disease (Group 3)

Univariate and multivariable logistic regression analyses for pure NDKD (group 3) are presented in Table 3. In univariate analysis, several clinical and laboratory variables were significantly associated with pure NDKD. Lower creatinine (cOR 0.89, 95% CI 0.84–0.95, *p* < 0.001) and higher eGFR (cOR 1.01, 95% CI 1.00–1.02, *p* = 0.013) were associated with increased odds of group 3 classification. Lower potassium (cOR 0.69, 95% CI 0.52–0.92, *p* = 0.011) and lower phosphorus (cOR 0.88, 95% CI 0.77–1.00, *p* = 0.049) were also significant. Lower albumin (cOR 0.68, 95% CI 0.52–0.88, *p* = 0.004), lower fasting glucose (cOR 0.99, 95% CI 0.99–1.00, *p* = 0.001), and lower HbA1c (cOR 0.71, 95% CI 0.63–0.81, *p* < 0.001) were associated with pure NDKD. Higher cholesterol (cOR 1.00, 95% CI 1.00–1.01, *p* = 0.005), higher LDL (cOR 1.01, 95% CI 1.00–1.01, *p* = 0.004), and higher uric acid (cOR 1.12, 95% CI 1.01–1.25, *p* = 0.036) were also associated.

In multivariable analysis, several independent predictors of pure NDKD remained significant. BMI was inversely associated with group 3 (aOR 0.87, 95% CI 0.78–0.98, *p* = 0.019). Serum albumin remained a strong independent predictor (aOR 0.26, 95% CI 0.11–0.62, *p* = 0.003). HbA1c was independently associated with lower odds of pure NDKD (aOR 0.41, 95% CI 0.28–0.62, *p* < 0.001). IgA showed a positive association (aOR 1.01, 95% CI 1.00–1.01, *p* = 0.009). Hypertension remained inversely associated with pure NDKD (aOR 0.16, 95% CI 0.05–0.55, *p* = 0.004).

Overall, lower BMI, lower serum albumin, lower HbA1c, higher IgA levels, and absence of hypertension were independently associated with pure NDKD. These findings suggest that metabolic status, nutritional markers, and immunologic activity contribute to distinguishing pure NDKD from diabetic or mixed kidney disease.

## 4. Discussion

In this large biopsy-based cohort of patients with type 2 diabetes, NDKD was highly prevalent, with more than half of patients (81.3%) demonstrating pure NDKD (54.4%) and a substantial proportion exhibiting mixed lesions (26.7%). These findings suggest that kidney disease in patients with diabetes cannot be reliably presumed to be diabetic nephropathy based solely on clinical presentation. Compared with previously published studies [7], our cohort demonstrated a notably higher proportion of NDKD among patients with diabetes undergoing renal biopsy (81.3%). Because kidney biopsy is not routinely performed in all patients with diabetes and chronic kidney disease, the present cohort likely represents a clinically enriched population with atypical presentations suspicious for NDKD. Therefore, both the prevalence estimates and the identified predictors observed in this study may have been influenced by biopsy indication bias and referral bias inherent to tertiary-care biopsy cohorts. Several factors may account for this difference. First, the threshold for performing renal biopsy in our clinical setting may be lower, particularly in patients presenting with atypical features, leading to a higher likelihood of detecting NDKD. Second, referral bias inherent to a tertiary care center may enrich the study population with more complex or diagnostically uncertain cases [10], thereby increasing the prevalence of non-diabetic etiologies. Third, differences in patient selection criteria, including the exclusion of transplant recipients and non-diagnostic biopsies, may have further contributed to the observed distribution. In addition, regional variations in disease spectrum and underlying etiologies of kidney disease may also play a role [11]. Collectively, these factors may explain the relatively high prevalence of NDKD observed in our cohort. The high burden of NDKD observed in our study highlights the limitations of conventional diagnostic assumptions and reinforces the important role of renal biopsy in establishing an accurate diagnosis.

In addition to its high prevalence, several readily available clinical and laboratory parameters were associated with biopsy-proven NDKD. Metabolic markers, particularly HbA1c, along with BMI and lipid profile, were associated with disease classification. Renal parameters, including urinary protein indices and serum albumin, also contributed to distinguishing NDKD from DN. These findings suggest that commonly used clinical markers of glycemic exposure and kidney injury do not uniformly reflect the underlying pathology and highlight the heterogeneity of kidney disease in patients with diabetes.

One notable finding was the heterogeneous association between glycemic control and underlying kidney pathology. Higher HbA1c was associated with NDKD in the combined analysis, whereas lower HbA1c was associated with pure NDKD. Several factors may explain this apparently discordant relationship. First, the pathological heterogeneity introduced by mixed lesions likely played an important role. In the primary analysis, patients with mixed DN and NDKD were included within the NDKD group. These patients may still retain substantial diabetic metabolic burden and chronic hyperglycemic exposure despite the coexistence of superimposed non-diabetic kidney disease, thereby contributing to relatively higher HbA1c levels in the combined analysis. In contrast, pure NDKD may occur in patients whose renal pathology is less directly driven by chronic hyperglycemia or diabetic microvascular injury [8,11,12], which may explain the association with relatively lower HbA1c levels. In addition, selection bias inherent to biopsy-based cohorts may further influence these associations, as patients with atypical clinical presentations are more likely to undergo renal biopsy regardless of glycemic status [7,10,11]. Collectively, these findings highlight the heterogeneity of kidney disease in patients with diabetes and suggest that HbA1c alone should not be interpreted as a definitive discriminator between diabetic nephropathy and NDKD.

Immunologic features further distinguished NDKD from diabetic nephropathy in our cohort [8]. Patients with NDKD demonstrated a higher prevalence of immunologic abnormalities [11], including anti–ds DNA positivity, reduced complement levels, and elevated immunoglobulin A [13], findings that are consistent with prior biopsy-based studies showing a higher burden of immune-mediated glomerular diseases among patients with diabetes. These abnormalities likely reflect the underlying pathophysiology of NDKD [13,14], in which immune complex deposition, complement activation, and inflammatory processes play central roles, as seen in conditions such as lupus nephritis and IgA nephropathy. In contrast, DN is predominantly driven by chronic hyperglycemia–induced metabolic and hemodynamic injury, with relatively limited involvement of immune-mediated mechanisms [12]. Importantly, the presence of immunologic signals may provide clinically useful clues to differentiate NDKD from diabetic nephropathy in patients with diabetes. In particular, complement consumption, autoantibody positivity, and elevated immunoglobulin levels may suggest an alternative or superimposed immune-mediated process, thereby supporting consideration of renal biopsy [10]. These findings underscore the value of integrating immunologic markers into the clinical evaluation of diabetic patients with kidney disease, especially in cases with atypical presentations.

To facilitate clinical translation of our findings, we developed a conceptual decision algorithm integrating key clinical, laboratory, and immunologic features associated with underlying kidney pathology (Supplementary Figure S2). This algorithm highlights how commonly available parameters, particularly HbA1c, proteinuria, renal function, and immunologic markers, may be combined to stratify the likelihood of diabetic nephropathy, mixed lesions, or NDKD. Notably, the framework emphasizes the identification of atypical features—such as hematuria, hypoalbuminemia, or positive autoimmune serology—which have been consistently associated with NDKD in prior studies and in our analysis. Importantly, this algorithm is not intended as a validated diagnostic tool but rather as a clinically oriented framework to support decision-making regarding renal biopsy. Given the inherent selection bias in biopsy-based cohorts and the heterogeneity of kidney disease in patients with diabetes, prospective validation in independent populations is warranted. Nevertheless, this approach underscores the potential value of integrating metabolic, renal, and immunologic domains to improve risk stratification and guide individualized evaluation in patients with diabetes and kidney disease.

This study has several strengths. The use of a large biopsy-proven cohort minimizes misclassification and provides a high level of diagnostic accuracy. The comprehensive collection of clinical, metabolic, immunologic, and urinary data enabled a multidimensional evaluation of predictors. In addition, the inclusion of both mixed lesions and pure NDKD allowed for a more nuanced assessment of disease heterogeneity. The real-world nature of the dataset further enhances the generalizability of our findings.

This study also has limitations. Important diabetes-related variables, including duration of diabetes, diabetic retinopathy, diabetic neuropathy, smoking status, and cardiovascular complications, were not consistently available because of the retrospective nature of the study and the long study period. Detailed medication exposure data, including renin–angiotensin system inhibitors, sodium–glucose cotransporter-2 inhibitors, mineralocorticoid receptor antagonists, and GLP-1 receptor agonists, were not consistently available because of the retrospective design and referral-center nature of the study. However, because of the retrospective design and the long study period spanning different treatment eras, detailed medication exposure data were not consistently available across the entire cohort. In addition, as our institution is a tertiary referral center, medication histories from outside hospitals were not always completely captured in the electronic health record system. Furthermore, these renoprotective therapies are primarily used for chronic kidney disease management and supportive care rather than for the treatment of the primary glomerular disease itself. Therefore, although these medications may modify renal functional parameters or proteinuria severity, they are unlikely to fundamentally alter the underlying pathological nature of glomerulonephritis identified on kidney biopsy.

Furthermore, detailed histopathological findings, including the degree of glomerulosclerosis, tubulointerstitial fibrosis, arteriolar hyalinosis, mesangial matrix expansion, and the presence of Kimmelstiel–Wilson nodules, were not uniformly documented across all biopsy reports and therefore could not be consistently analyzed. These pathological parameters may provide additional mechanistic and histological insights into the differences among disease groups. Despite these limitations, our study provides clinically relevant insights that may assist in identifying patients with diabetes who are more likely to have NDKD.

## 5. Conclusions

In this large biopsy-based cohort of patients with type 2 diabetes, NDKD was common and exhibited distinct clinical features compared with diabetic nephropathy. Several readily available clinical variables were associated with NDKD. These findings suggest that reliance on traditional assumptions may lead to misclassification and that integrated clinical assessment may help identify patients who are more likely to benefit from renal biopsy.

## Figures and Tables

**Table 1:** Baseline Characteristics Stratified by Kidney Disease Type.

Variable	DN (n = 124)	DN + NDKD (n = 179)	NDKD (n = 361)	*p*-Value	SMD
Demographics					
Age (years)	53.8 ± 12.0	56.7 ± 12.7	56.1 ± 15.3	0.175	0.17
Male sex (%)	60.5	60.9	54.6	0.284	0.12
BMI (kg/m^2^)	27.2 ± 4.6	26.7 ± 4.4	25.9 ± 4.7	0.081	0.28
Hematology					
WBC (×10^9^/L)	8.07 ± 2.61	8.00 ± 2.80	8.43 ± 3.96	0.333	0.10
Neutrophil (%)	69.2 ± 9.8	69.0 ± 10.1	69.4 ± 12.5	0.942	0.02
Lymphocyte (%)	21.4 ± 8.7	21.9 ± 9.5	21.8 ± 10.6	0.911	0.04
Hemoglobin (g/dL)	10.9 ± 2.5	10.9 ± 2.4	11.9 ± 2.5	<0.001	0.40
Platelet (×10^9^/L)	257 ± 102	251 ± 80	253 ± 94	0.84	0.04
Renal Function					
Creatinine (mg/dL)	3.95 ± 3.50	3.21 ± 2.23	2.68 ± 2.75	<0.001	0.42
eGFR (mL/min/1.73 m^2^)	35.3 ± 31.9	33.1 ± 29.4	49.5 ± 37.2	<0.001	0.45
BUN (mg/dL)	49.3 ± 31.1	42.5 ± 23.6	38.5 ± 29.7	0.002	0.33
Biochemistry					
ALT (U/L)	28 ± 30	23 ± 16	27 ± 26	0.199	0.04
AST (U/L)	28.6 ± 22.5	26.7 ± 14.0	28.7 ± 19.9	0.635	0.01
Sodium (mmol/L)	138.5 ± 4.0	139.2 ± 3.5	139.0 ± 4.3	0.366	0.12
Potassium (mmol/L)	4.3 ± 0.7	4.2 ± 0.7	4.1 ± 0.6	0.008	0.30
Calcium (mg/dL)	8.4 ± 0.7	8.3 ± 0.8	8.4 ± 0.8	0.106	0.02
Phosphorus (mg/dL)	4.7 ± 2.0	4.3 ± 1.2	4.4 ± 1.4	0.120	0.15
LDH (U/L)	237 ± 73	261 ± 80	253 ± 123	0.355	0.16
Albumin (g/dL)	3.5 ± 0.6	3.1 ± 0.7	3.3 ± 0.8	0.002	0.38
Total protein (g/dL)	6.3 ± 0.9	6.0 ± 1.2	6.1 ± 1.3	0.217	0.15
Metabolic Profile					
Fasting glucose (mg/dL)	140 ± 58.7	135 ± 53.1	113 ± 34.9	<0.001	0.51
HbA1c (%)	7.3 ± 1.6	7.1 ± 1.6	6.2 ± 1.0	<0.001	0.75
Cholesterol (mg/dL)	194 ± 62.5	218 ± 83	222 ± 93.6	0.017	0.33
LDL (mg/dL)	109.5 ± 48.7	131 ± 70.7	134 ± 83.1	0.012	0.31
HDL (mg/dL)	47.8 ± 16.9	50.6 ± 21	50.4 ± 20.2	0.623	0.14
Triglycerides (mg/dL)	220 ± 180	259 ± 445	250 ± 507	0.769	0.09
Uric acid (mg/dL)	6.8 ± 1.9	6.9 ± 1.9	7.5 ± 2.3	0.003	0.33
Immunologic Data					
ANA (%)	29.8	28.5	29.6	0.959	0.01
ANCA (%)	3.9	3.2	10.3	0.008	0.28
dsDNA (%)	18.2	18.4	39.2	<0.001	0.45
C3 (mg/dL)	119.7 ± 25.1	118.8 ± 29.1	111.7 ± 34.4	0.041	0.25
C4 (mg/dL)	39 ± 12	37 ± 13	32 ± 13	<0.001	0.50
IgG (mg/dL)	1082 ± 338	1071 ± 672	1083 ± 642	0.985	0.00
IgA (mg/dL)	283 ± 107	326 ± 153	317 ± 170	0.153	0.22
Urine Profile					
UACR (mg/g)	4642 ± 3929	5232 ± 4145	3761 ± 4924	0.029	0.20
UPCR (mg/g)	5841 ± 6229	7491 ± 6128	4897 ± 6696	<0.001	0.35
Hematuria (%)	66.4	62.9	67.9	0.511	0.03
Comorbidities					
Hypertension (%)	79.8	74.3	64.5	0.002	0.34
Lupus (%)	6.5	2.8	16.6	<0.001	0.45
Malignancy (%)	12.1	12.8	12.5	0.987	0.01

Abbreviations: BMI, body mass index; WBC, white blood cell count; eGFR, estimated glomerular filtration rate; BUN, blood urea nitrogen; ALT, alanine aminotransferase; AST, aspartate aminotransferase; LDH, lactate dehydrogenase; HbA1c, glycated hemoglobin; LDL, low-density lipoprotein cholesterol; HDL, high-density lipoprotein cholesterol; UACR, urine albumin-to-creatinine ratio; UPCR, urine protein-to-creatinine ratio; ANA, antinuclear antibody; ANCA, anti-neutrophil cytoplasmic antibody; dsDNA, anti-double-stranded DNA; Ig, immunoglobulin. SMD (standardized mean difference) was calculated between DN and NDKD groups (group 1 vs. group 3). An SMD > 0.3 was considered clinically meaningful.

**Table 2:** Logistic Regression for NDKD (Groups 2 and 3 vs. DN).

Variable	cOR (95% CI)	*p*-Value	aOR (95% CI)	*p*-Value
Demographics				
Age	0.965 (0.945–0.985)	<0.001	0.934 (0.881–0.990)	0.021
Male sex	0.912 (0.621–1.338)	0.635	—	—
BMI	1.048 (1.003–1.095)	0.037	0.811 (0.695–0.946)	0.008
Hematology				
Hemoglobin	0.854 (0.799–0.912)	<0.001	—	—
WBC	1.018 (0.969–1.069)	0.471	—	—
Platelet	0.999 (0.997–1.001)	0.295	—	—
Renal Function				
Creatinine	1.115 (1.062–1.171)	<0.001	—	—
eGFR	0.986 (0.981–0.992)	<0.001	—	—
BUN	1.007 (1.002–1.012)	0.006	—	—
Biochemistry				
Sodium	0.982 (0.943–1.023)	0.395	—	—
Potassium	1.411 (1.108–1.798)	0.005	—	—
Albumin	0.772 (0.616–0.968)	0.024	—	—
Metabolic				
Fasting glucose	1.013 (1.009–1.018)	<0.001	—	—
HbA1c	2.026 (1.636–2.508)	<0.001	3.675 (1.75–7.718)	0.001
LDL	0.996 (0.993–0.999)	0.009	0.979 (0.966–0.992)	0.002
Uric acid	0.872 (0.802–0.948)	0.001	—	—
Immunologic				
ANCA	0.313 (0.142–0.692)	0.003	—	—
dsDNA	0.348 (0.223–0.541)	<0.001	—	—
C3	1.008 (1.002–1.014)	0.012	—	—
C4	1.038 (1.019–1.058)	<0.001	—	—
Urine				
UACR	1.0003 (1.0001–1.0005)	<0.001	1.00038 (1.0001–1.001)	0.007
UPCR	1.0001 (1.0000–1.0002)	0.041	0.99984 (0.9997–1.0000)	0.061
Hematuria	0.915 (0.620–1.351)	0.657	—	—
Comorbidities				
Hypertension	1.795 (1.252–2.572)	0.001	—	—
Lupus	0.225 (0.130–0.391)	<0.001	—	—
Asthma	0.466 (0.227–0.957)	0.037	—	—

Abbreviations: cOR, crude odds ratio; aOR, adjusted odds ratio; CI, confidence interval; BMI, body mass index; eGFR, estimated glomerular filtration rate; BUN, blood urea nitrogen; HbA1c, glycated hemoglobin; LDL, low-density lipoprotein cholesterol; UACR, urine albumin-to-creatinine ratio; UPCR, urine protein-to-creatinine ratio; ANCA, anti-neutrophil cytoplasmic antibody; dsDNA, anti-double-stranded DNA.

**Table 3:** Logistic Regression for Pure NDKD (Group 3).

Variable	cOR (95% CI)	*p*-Value	aOR (95% CI)	*p*-Value
Demographics				
Age	1.009 (0.995–1.023)	0.209	—	—
BMI	0.953 (0.913–0.995)	0.029	0.871 (0.776–0.978)	0.019
Renal Function				
Creatinine	0.888 (0.835–0.945)	<0.001	—	—
eGFR	1.008 (1.002–1.015)	0.013	—	—
Biochemistry				
Potassium	0.689 (0.516–0.921)	0.011	—	—
Phosphorus	0.879 (0.773–0.999)	0.049	—	—
Albumin	0.676 (0.517–0.883)	0.004	0.26 (0.108–0.624)	0.003
Metabolic				
Fasting glucose	0.993 (0.989–0.998)	0.001	—	—
HbA1c	0.712 (0.630–0.805)	<0.001	0.414 (0.277–0.618)	<0.001
Cholesterol	1.004 (1.001–1.007)	0.005	—	—
LDL	1.006 (1.002–1.010)	0.004	—	—
Uric acid	1.121 (1.007–1.247)	0.036	—	—
Immunologic				
dsDNA	2.154 (1.154–4.019)	0.016	—	—
C4	0.969 (0.953–0.986)	<0.001	—	—
IgA	1.002 (1.000–1.004)	0.048	1.006 (1.001–1.010)	0.009
Urine				
Hematuria (RBC)	1.015 (1.000–1.031)	0.047	—	—
Comorbidities				
Hypertension	0.531 (0.331–0.852)	0.009	0.16 (0.047–0.548)	0.004
Asthma	4.357 (1.033–18.36)	0.045	—	—

Abbreviations: cOR, crude odds ratio; aOR, adjusted odds ratio; CI, confidence interval; BMI, body mass index; eGFR, estimated glomerular filtration rate; HbA1c, glycated hemoglobin; LDL, low-density lipoprotein cholesterol; IgA, immunoglobulin A.

## Data Availability

The data supporting the findings of this study cannot be publicly shared due to institutional regulations and privacy.

## Supplementary Materials

The following supporting information can be downloaded at: https://www.mdpi.com/article/10.3390/jcm15114346/s1, Table S1. Baseline Characteristics (Median [IQR]); Figure S1. Standardized mean differences (SMDs) of baseline variables between patients with diabetic nephropathy (DN) (group 3) and non-diabetic kidney disease (NDKD) (group 1); Figure S2. Clinical decision algorithm for predicting kidney disease pathology in patients with type 2 diabetes undergoing renal biopsy.

## Abbreviations

AKI	acute kidney injury
ALT	alanine aminotransferase
ANA	antinuclear antibody
ANCA	anti-neutrophil cytoplasmic antibody
aOR	adjusted odds ratio
AST	aspartate aminotransferase
BMI	body mass index
BUN	blood urea nitrogen
CI	confidence interval
CKD	chronic kidney disease
cOR	crude odds ratio
DM	diabetes mellitus
DN	diabetic nephropathy
dsDNA	anti-double-stranded DNA
eGFR	estimated glomerular filtration rate
ESKD	end-stage kidney disease
ETL	extract–transform–load
HbA1c	glycated hemoglobin
HDL	high-density lipoprotein cholesterol
Ig	immunoglobulin
IgA	immunoglobulin A
IgE	immunoglobulin E
IgG	immunoglobulin G
IgM	immunoglobulin M
LDH	lactate dehydrogenase
LDL	low-density lipoprotein cholesterol
MDRD	Modification of Diet in Renal Disease
NDKD	non-diabetic kidney disease
SGLT2i	sodium–glucose cotransporter 2 inhibitors
SMD	standardized mean difference
UACR	urine albumin-to-creatinine ratio
UPCR	urine protein-to-creatinine ratio
WBC	white blood cell count

## References

[1] GBD Chronic Kidney Disease Collaboration (2020). Global, regional, and national burden of chronic kidney disease, 1990–2017: A systematic analysis for the Global Burden of Disease Study 2017. Lancet.

[2] Jha V., Garcia-Garcia G., Iseki K., Li Z., Naicker S., Plattner B., Saran R., Wang A.Y., Yang C.W. (2013). Chronic kidney disease: Global dimension and perspectives. Lancet.

[3] Yang W.-C., Hwang S.-J., Nephrology T.S.O. (2008). Incidence, prevalence and mortality trends of dialysis end-stage renal disease in Taiwan from 1990 to 2001: The impact of national health insurance. Nephrol. Dial. Transplant..

[4] Ou S.-M., Chao C.-T., Lin M.-Y., Hsu C.-C., Lin M.-H., Chou C.-L., Hsu Y.-H., Hwang S.-J., Wu M.-S., Wu M.-Y. (2024). Summary of the 2024 Annual Report on Kidney Disease in Taiwan. Acta Nephrol..

[5] Johansen K.L., Gilbertson D.T., Li S., Li S., Liu J., Roetker N.S., Ku E., Schulman I.H., Greer R.C., Chan K. (2024). US Renal Data System 2023 Annual Data Report: Epidemiology of Kidney Disease in the United States. Am. J. Kidney Dis..

[6] Kidney Disease: Improving Global Outcomes (KDIGO) Diabetes Work Group (2022). KDIGO 2022 Clinical Practice Guideline for Diabetes Management in Chronic Kidney Disease. Kidney Int..

[7] Anders H.J., Huber T.B., Isermann B., Schiffer M. (2018). CKD in diabetes: Diabetic kidney disease versus nondiabetic kidney disease. Nat. Rev. Nephrol..

[8] Sharma S.G., Bomback A.S., Radhakrishnan J., Herlitz L.C., Stokes M.B., Markowitz G.S., D’Agati V.D. (2013). The modern spectrum of renal biopsy findings in patients with diabetes. Clin. J. Am. Soc. Nephrol..

[9] Sreedharan S., M. K.M., K. B.V., Raju N. (2026). Non-diabetic Kidney Disease in Type 2 Diabetes: From Kidney Biopsy to Precision Medicine. Cureus.

[10] Hull K.L., Adenwalla S.F., Topham P., Graham-Brown M.P. (2022). Indications and considerations for kidney biopsy: An overview of clinical considerations for the non-specialist. Clin. Med. (Lond.).

[11] Fiorentino M., Bolignano D., Tesar V., Pisano A., Biesen W.V., Tripepi G., D’Arrigo G., Gesualdo L. (2017). Renal biopsy in patients with diabetes: A pooled meta-analysis of 48 studies. Nephrol. Dial. Transpl..

[12] Tervaert T.W., Mooyaart A.L., Amann K., Cohen A.H., Cook H.T., Drachenberg C.B., Ferrario F., Fogo A.B., Haas M., de Heer E. (2010). Pathologic classification of diabetic nephropathy. J. Am. Soc. Nephrol..

[13] Wyatt R.J., Julian B.A. (2013). IgA nephropathy. N. Engl. J. Med..

[14] Anders H.J., Saxena R., Zhao M.H., Parodis I., Salmon J.E., Mohan C. (2020). Lupus nephritis. Nat. Rev. Dis. Primers.

