# Supplementary material for: Clinical Predictors of Non-Diabetic Kidney Disease in Patients with Diabetes: Insights from a Biopsy-Proven Cohort
Source: J Clin Med. 2026 Jun 4;15(11):4346. doi: 10.3390/jcm15114346 (PMC13257530; doi:10.3390/jcm15114346)

Supplementary Table S1. Baseline Characteristics (Median [IQR])

Variable	DN (n=124)	DN+NDKD (n=179)	NDKD (n=361)	p-value
**Demographics**				
Age (years)	53 (46–64)	57 (47–65)	59 (45–66)	0.09
BMI (kg/m²)	26.9 (24.3–29.6)	25.6 (23.5–28.7)	25.3 (22.8–28.7)	0.033
**Hematology**				
WBC (×10⁹/L)	7.63 (6.20–9.22)	7.65 (6.20–9.70)	7.46 (6.05–9.73)	0.98
Neutrophil (%)	69.2 (62.3–76.6)	69.7 (62.5–75.1)	68.1 (60.4–79.5)	0.971
Lymphocyte (%)	21.2 (14.7–28.0)	20.3 (15.4–27.1)	22.1 (13.0–29.0)	0.941
Hemoglobin (g/dL)	10.2 (9.3–12.7)	10.5 (9.0–12.7)	12.0 (10.0–13.6)	<0.001
Platelet (×10⁹/L)	237 (188–314)	245 (193–296)	242 (190–310)	0.99
**Renal Function**				
Creatinine (mg/dL)	2.83 (1.32–5.11)	2.60 (1.61–4.20)	1.63 (0.98–3.10)	<0.001
eGFR (mL/min/1.73m²)	20.1 (11.4–55.6)	25.0 (14.7–44.7)	43.3 (19.5–73.8)	<0.001
BUN (mg/dL)	41 (27–64.5)	38 (25–52)	28 (18–50)	<0.001
**Biochemistry**				
ALT (U/L)	19 (14–32)	19 (14–27)	20 (13–28)	0.647
AST (U/L)	22 (16–31)	23 (18–31)	24 (19–31)	0.624
Sodium (mmol/L)	139 (137–141)	140 (137–141)	140 (137–142)	0.244
Potassium (mmol/L)	4.3 (3.8–4.8)	4.2 (3.8–4.7)	4.1 (3.8–4.5)	0.007
Phosphorus (mg/dL)	4.2 (3.6–5.0)	4.1 (3.6–4.9)	4.0 (3.5–4.8)	0.360
Calcium (mg/dL)	8.3 (7.8–8.9)	8.2 (7.8–8.8)	8.4 (7.9–8.9)	0.101
LDH (U/L)	226 (185–284)	243 (208–302)	224 (189–276)	0.050
Albumin (g/dL)	3.5 (3.0–3.9)	3.1 (2.6–3.6)	3.4 (2.6–4.0)	0.0017

Total protein (g/dL)	6.4 (5.7–6.9)	5.9 (5.2–6.8)	6.2 (5.2–7.1)	0.104
**Metabolic Profile**				
Fasting glucose (mg/dL)	127 (99–157)	122 (101–154)	104 (91–126)	<0.001
HbA1c (%)	6.9 (6.1–8.0)	6.7 (6.1–7.6)	6.0 (5.6–6.5)	<0.001
Cholesterol (mg/dL)	177 (148–234)	204 (156–257)	206 (163–259)	0.025
LDL (mg/dL)	102 (71–132)	112 (82–162)	115 (81–156)	0.030
HDL (mg/dL)	44 (38.5–53)	46 (37–60)	45 (37–61)	0.887
Triglycerides (mg/dL)	182 (101–273)	178 (125–253)	166 (108–243)	0.489
Uric acid (mg/dL)	6.7 (5.5–7.9)	6.9 (5.7–7.9)	7.1 (5.9–8.7)	0.020
**Immunologic Data**				
C3 (mg/dL)	119.2 (101.7–136.5)	116.7 (97.9–137.7)	110.8 (90.8–136.1)	0.093
C4 (mg/dL)	38 (33–46)	35 (29–45)	31 (24–40)	<0.001
IgG (mg/dL)	1071 (812–1292)	905 (678–1305)	1018 (710–1298)	0.277
IgA (mg/dL)	282 (197–358)	302 (233–383)	288 (212–395)	0.294
IgM (mg/dL)	70 (58–102)	81 (54–113)	88 (58–132)	0.135
IgE (mg/dL)	98 (27–258)	76 (28–299)	92 (29–251)	0.991
**Urine Profile**				
UACR (mg/g)	4233 (2016–6099)	4843 (1799–7544)	1861 (666–5216)	<0.001
UPCR (mg/g)	4421 (947–9424)	6890 (2178–11543)	2549 (880–6454)	<0.001
Urine WBC (cells/HPF)	0 (0–2)	0 (0–2)	0 (0–5)	0.074
Hematuria RBC (cells/HPF)	2 (0–5)	2 (0–5)	3 (0–10)	0.001

Abbreviations:

IQR, interquartile range; BMI, body mass index; WBC, white blood cell count; eGFR, estimated glomerular filtration rate; BUN, blood urea nitrogen; ALT, alanine aminotransferase; AST, aspartate aminotransferase; LDH, lactate dehydrogenase; HbA1c, glycated hemoglobin; LDL, low-density lipoprotein cholesterol; HDL, high-density lipoprotein cholesterol; UACR, urine albumin-to-creatinine ratio; UPCR, urine protein-to-creatinine ratio; ANA, antinuclear antibody; ANCA, anti-neutrophil cytoplasmic antibody; dsDNA, anti–double-stranded DNA; Ig, immunoglobulin.

Figure S1. Standardized mean differences (SMDs) of baseline variables between patients with diabetic nephropathy (DN) (group 3) and non-diabetic kidney disease (NDKD) (group 1).

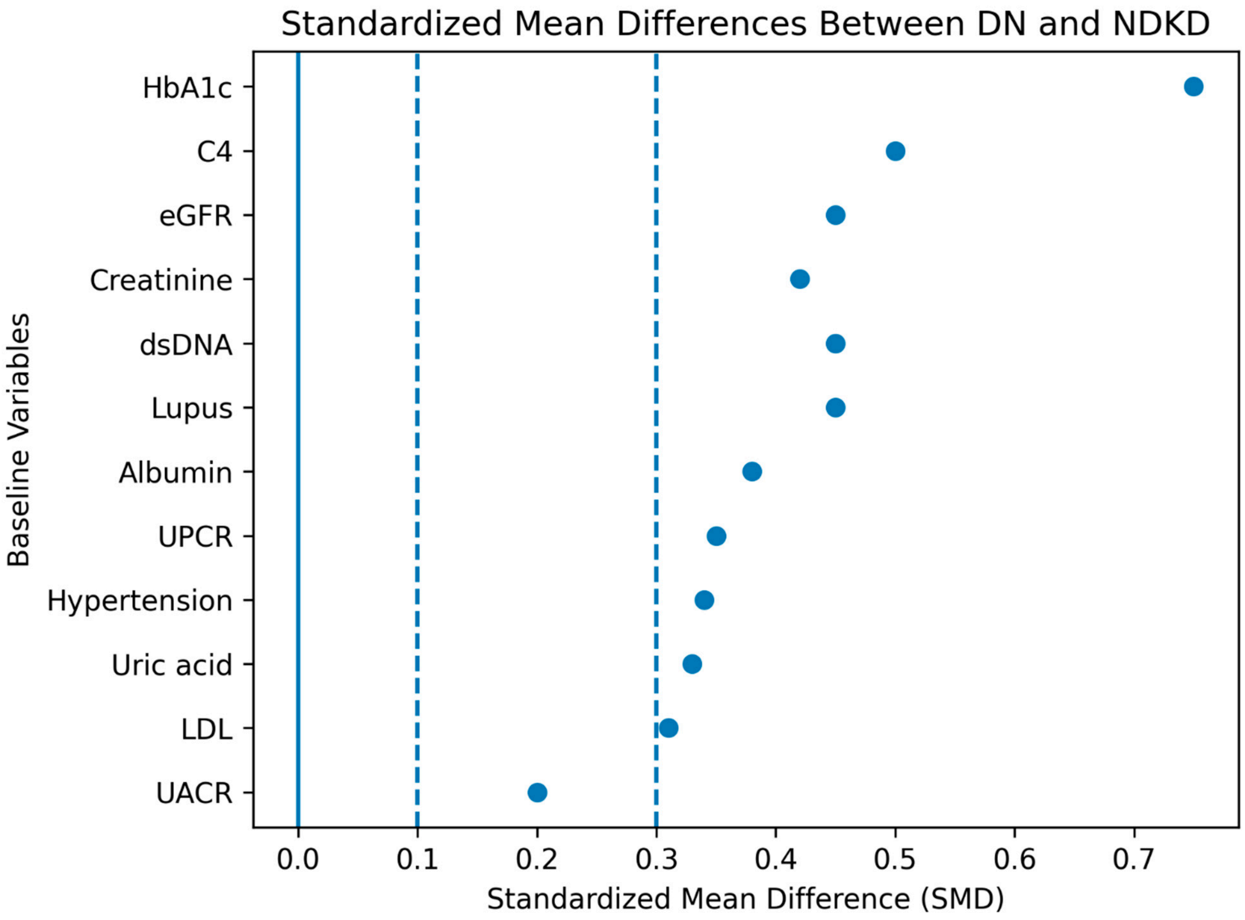

Variables are ordered by effect size. The vertical solid line at 0 indicates no difference. Dashed lines at 0.1 and 0.3 represent thresholds for small and clinically meaningful differences, respectively.

Figure S2. Clinical decision algorithm for predicting kidney disease pathology in patients with type 2 diabetes undergoing renal biopsy

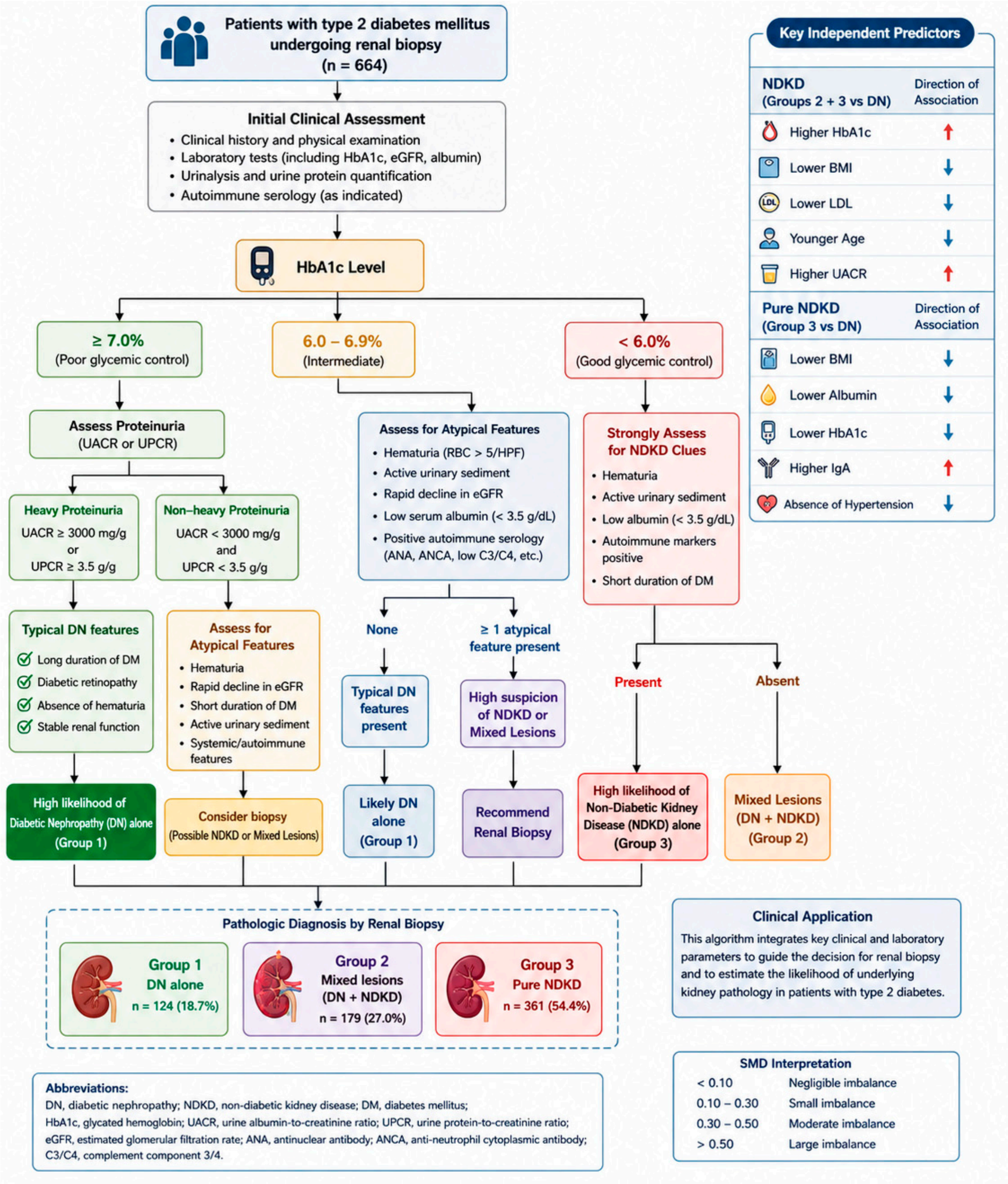

Supplement: Supplementary file 1 [file jcm-15-04346-s001.zip › jcm-4327569-supplementary.pdf]
